# Low Expression of Mfn2 Is Associated with Mitochondrial Damage and Apoptosis of Ovarian Tissues in the Premature Ovarian Failure Model

**DOI:** 10.1371/journal.pone.0136421

**Published:** 2015-09-01

**Authors:** Wenqi Chen, Xiaoyan Xu, Lingjuan Wang, Ge Bai, Wenpei Xiang

**Affiliations:** 1 Family Planning Research Institute, Tongji Medical College, Huazhong University of Science and Technology, Wuhan, 430030, China; 2 Department of Obstetrics and Gynecology, Tongji Hospital, Tongji Medical College, Huazhong University of Science and Technology, Wuhan, 430030, China; East Carolina University, UNITED STATES

## Abstract

**Background:**

This study aimed to construct a working model for detecting the mitochondrial damage and expression of *Mfn2*. It furthermore explored the pathogenesis of premature ovarian failure (POF) induced by cisplatin.

**Method:**

Forty young female mice were divided randomly into two groups. The first was the treatment group intraperitoneally administered cisplatin (1.5mg/kg). The untreated control group was likewise injected with physiological saline for 10 days. One month later, we observed the ovarian weight and morphological changes, particularly the development of follicles and concentration of sex hormones. Immunohistochemistry and western blotting were used to measure the two groups. We later evaluated ovarian cell apoptosis with TUNEL and analyzed Bcl-2 and Bax levels. We used transmission electron microscopy in order to observe the ultrastructure of ovarian cells. The phosphomolybdic acid colorimetric method was used to measure the ATP content in the ovarian tissue. Finally, the mitochondrial membrane potential of ovarian cells was detected with JC-1 dye.

**Results:**

The cisplatin resulted in a decline of body weight, reduced ovarian weight significantly, and resulted in disorders of the extrous cycle. The follicles’ number decreased within the tissue’s stromal hyperplasia. Moreover, E_2_ levels were reduced, and elevated gonadotropin levels were observed. However, Mfn2 was present in the cell’s cytoplasm in both groups. Nevertheless, the Mfn2 levels and the expression of *Bcl-2* were significantly decreased (*p*<0.05), but the expression of *Bax* and the apoptosis index (AI) was increased. In addition, the ATP levels (35.2 ±5.7μmol/g) of the control group were significantly higher (13.5 ± 3.8 μmol/g). Lastly, an obvious impairment of mitochondrial function and structure was observed.

**Conclusion:**

The intreperitoneal injection of cisplatin, when administered for 10 days, establishes a POF model. Thus, the above results suggest that lower expression of *Mfn2* may be involved in the mechanism of premature ovarian failure by affecting both the mitochondria’s energy metabolism and its apoptosis. This decides the termination of the follicles’ development.

## Introduction

Premature ovarian failure (POF) is a disease defined as premature menopause, or the cessation of ovarian function before the age of 40. This is characterized by amenorrhea, high gonadotropin levels, and low estrogen levels [1]. The etiology of POF involves immunologic, genetic, metabolic, environmental and iatrogenic factors. Although its underlying mechanisms have been extensively investigated, the pathogenesis of POF itself remains unclear.

Apoptosis is a form of cell death triggered by a variety of intracellular and extracellular signals, including mitochondrial pathways. Granulose cells (GC), a kind of functional cell, are able to synthesize many active peptides, and are involved in the synthesis of estrogen and progesterone [2,3]. Follicular atresia is the apoptosis of ovarian granular cells and oocytes. Most studies reveal that the speeding up of follicular atresia has become a key component of POF [4,5]. Thus, the apoptosis of GC becomes an initiating factor and plays a key role in the occurrence of POF [6].

Mitochondria, the main provider of cell energy, plays an important role in the process of cell apoptosis, it is a kind of abundant particle in granular cells, regulates cell metabolism, cell cycle, and cell signal transduction [7]. Many studies prove that mitochondrial change [8, 9] is related to the pathogenesis of POF. Mitochondrial dysfunction therefore leads to pathological changes, disrupts the production of normal ATP levels, and increases the number of reactive oxygen species (ROS), and then affects granular cell function as well as the development of oocytes. This continues even unto the point of inducing cell death [10, 11]. Therefore, there is obviously a close relationship between mitochondria and women aging; although, the underlying mechanism as of yet remains to be investigated.

Mitofusin2 (Mfn2), a conserved dynamin-like GTPase located in the outer membrane of the mitochondria, affects the structure and function of mitochondria by regulating its process of fusion and fission [12, 13]. As many studies have confirmed that Mfn2 sustains normal mammalian cellular function by regulating its respiratory chain, mitochondrial membrane potential, and cellular metabolism and apoptosis [14, 15], lower expression of *Mfn2* can increase the stress of the endoplasmic reticulum, which may result in granulose cell apoptosis [16, 17]. Mfn2 plays an important role in maintaining the integrity of the mitochondrial DNA [18] and its abnormality may affect the function of granulose cells and the development of oocytes through mitochondrial oxidative phosphorylation. Our research results have shown that lower expression of *Mfn2* affects embryonic development by regulating its mitochondrial function and inducing apoptosis [19, 20]. However, whether Mfn2 has an effect on POF by regulating apoptosis of GC remains unknown. This study has investigated *Mfn2* expression in POF of mice's ovarian tissue and has aimed to identify the relationship between Mfn2 and POF and has revealed the important role of Mfn2 in the process of POF.

## Material and Methods

### POF model construction

Following approval of the Animal Research Center of the Huazhong University of Science and Technology as well as many studies based on the successful POF model [21–22], our POF models were constructed in female KM (4~6 weeks, 28~30g) mice. The mice were left to adapt for 3~5 days after their purchase. We obtained their weight and then randomly divided them into two groups: one cisplatin treatment group, and the other the untreated control group. The treatment group (cisplatin) received daily intraperitoneal (i.p.) injections of cisplatin (Sigma, USA) (1.5mg/kg) for 10 days. The control group (control) received equivalent doses of normal saline for 10 days. Blood from each group was collected after one month of treatment, via direct cardiac puncture. The serum of the two groups was isolated and stored at -80°C, to be used for enzyme-linked immunosorbent assay (ELISA). The estradiol (E2) and follicle-stimulating hormone (FSH) plasma levels were measured using the ELISA kit (life science and technology Co, Wuhan). The mice's ovarian tissues of both groups were preserved within liquid nitrogen immediately after, which was fixed with a 4% paraformaldehyde solution.

### Immunohistochemistry technique

The ovarian tissue was fixed overnight in the 4% paraformaldehyde at room temperature. Then after dehydration they were embedded in paraffin, made transparent, and then cut into 4-μm-thick serial sections. They were then stained with HE (Boster, Wuhan) for general histomorphometric analysis. Our immunohistochemical technique was performed in accordance with the instructions on the HRP Conjugated anti-Rabbit IgG SABC Kit (Boster, Wuhan). Slides were dewaxed and rehydrated with xylol and ethanol. Endogenous peroxidase was quenched with a 3% H_2_O_2_ solution and an nonspecific staining’ sealing mixed with goat serum (Boster, Wuhan), the slides were incubated overnight at 4°C with rabbit polyclonal anti-mitofusin 2/Mfn2 antibody (Abcam, USA). PBS was used instead of the primary antibody, to be used as Negative control for each tissue section. The slides were then incubated with the biotinylated secondary antibody—polyclonal goat anti-rabbit antibody (Jackson ImmunoResearch, USA), for one hour, at 37°C incubation with SABC complex (Boster, Wuhan) and DAB Staining (Boster, Wuhan) were followed by the PBS washing. Both groups were processed under identical conditions.

### Western blotting analysis

The proteins (30μg) described above were then separated by SDS-PAGE, and transferred to PVDF membranes (Invitrogen, USA). Nonspecific binding was blocked by using of 5% nonfat milk for 1 h at 37°C. The nitrocellulose membranes were then hybridized overnight at 4°C with the rabbit polyclonal anti-Mfn2 antibody (Abcam, USA), the rabbit polyclonal anti-Bcl-2 antibody (Cell Signaling Technology, USA), the rabbit polyclonal anti-Bax antibody (Cell Signaling Technology, USA), and the rabbit polyclonal anti-β-actin antibody (Santa cruz Biotechnology Inc, USA) in the Primary Antibody Dilution Buffer. After washing four times with TBS-T, each time for 10 minutes at 37°C the membranes were incubated with goat anti-rabbit horseradish peroxidase (HRP)-conjugated secondary antibody for 1 h at 37°C (AntGene, USA). Finally, the immunoreactive bands were detected by use of the Enhanced Chimio-Luminescent system (Beyotime Institute of Biotechnology, China). Washing the bands four times in TBS-T. The band intensity was quantified by densitometry using the Quantity One 4.62 analysis software, and all results were normalized to β-actin signal intensity.

### RNA isolation, reverse transcription, and polymerase chain reaction (RT-PCR)

The ovarian tissues were taken out from the liquid nitrogen in order to extract RNA using 400μl Trizol according to the manufacturer’s instructions. 3μl RNA from each group was diluted with RNAase-free water, and was quantified using a Type 721 spectrophotometer at 260 nm. The cDNA was synthesized using RNA, oligo (dT) _18_ primers, and the First Strand cDNA Synthesis Kit (Fermentas, CA) according to the manufacturer’s protocol. The amplified detection of *Mfn2* and *β-actin* (housekeeping gene) mRNA expression was examined by Real-time Fluores-cence Quantitative PCR (Stratagene, USA) using All-in-One qPCR Mix Kit (Genecopoeia, USA). The primer's sequences are listed as follows: Mfn2 (110bp), F: 5’-CTTGAAGACACCCACAG-GAACA-3’, R: 5’-GGCCAGCACTTCGCTGATAC-3’; β-actin (266bp), F: 5’GTCCCTCACCCTCCCAAAAG-3’, R: 5’-GCTGCCTCAACACCTCAACCC-3’.

The Threshold Cycle (CT values) from real time PCR results was analyzed by the 2^-ΔΔCT^ method. Amplification was validated by running in 120 V× 25 min electrophoresis conditions and the images were acquired by means of the UVP gel imaging system.

### Measurement of mitochondrial membrane potential (ΔΨm)

Mitochondrial membrane potential was assessed by fluorescence microscopy after having used the JC-1 Mitochondrial Membrane Potential Assay Kit (Cayman Chemical Company, USA). The ovarian tissues (0.05g) were rinsed, cut, and ground with 500μl physiological saline. The homogenate was filtered using a mesh sieve, and was centrifuged in order to acquire single cell suspension. The cell precipitates were incubated in JC-1 fluorescent dye in a 5% CO_2_ incubator for 30 min at 37°C. The precipitate was then slowly washed with JC-1 working liquid three times. The mitochondrial membrane potential was analyzed by a fluorescence microscope using a 540/570 nm filter. The green signal of JC-1 was measured at 485/535 nm, whereas that of the red was measured at 590/610 nm.

### Detecting ATP levels

Tissues from all the groups were cut, ground, and mixed with boiling ddH_2_O to a final concentration of 10%. The homogenate was then boiled in water for another 10 min. The supernatant was used for the ATP assay kit (Nanjing Jiancheng Bioengineering Institute, China), after centrifugation at 3500 rpm for 15 min. The absorbance values of all samples were analyzed by UV/Visible Spectrophotometer (Perkin Elmer, USA) and the ATP content in the organization was calculated by using the general formula.

### TUNEL assay

Terminal-deoxynucleotidyl transferase-mediated nick end labeling (TUNEL) was performed using the Calbiochem TdT- FragEL DNA Fragmentation Detection Kit (Calbiochem, Germany), in order to detect apoptosis in the 4-μm-thick paraffined sections. The slides were then dewaxed, rehydrated, and washed in a 1×TBS buffer. The remaining reagent was added according to the manufacturer’s protocol. Meanwhile, all the negative controls were processed in the absence of TdT Labeling Reaction Mix and the TdT enzyme. As a side note, the apoptotic cells that usually contain the characteristic fragmented nuclear chromatin exhibited blue nuclear staining instead of the brown nuclear staining. The apoptosis index (AI) was analyzed using Image-pro plus 6.0 software, and calculated as (the number of TUNEL-positive nuclei in a zone)/(the number of total nuclei in the same zone) ×100%.

### Transmission electron microscopy (TEM)

The fresh ovarian tissues (5× 5 × 5 mm^3^) were immersed in a fixed liquid (Wuhan Google biological co., LTD., China) containing 2.5% glutaraldehyde for 10 min after being washed with sterile Physiological saline, and were then immediately sent to the ultrastructural pathological room of the Virus Research Institute in the China University of Science and Technology, in order to be subsequently operated upon. The cell ultrastructure was observed using a transmission electron microscope (FEI, Holland).

### Statistical analysis

The data was analyzed using software from the Statistical Program for Social Science (SPSS, Chicago, IL, USA), and every experiment was repeated a minimum of three times. The data is expressed in mean±standard error (x±s). Statistical significance was based on P<0.05.

## Results

### Cisplatin results in ovarian damage

**Fig 1:**
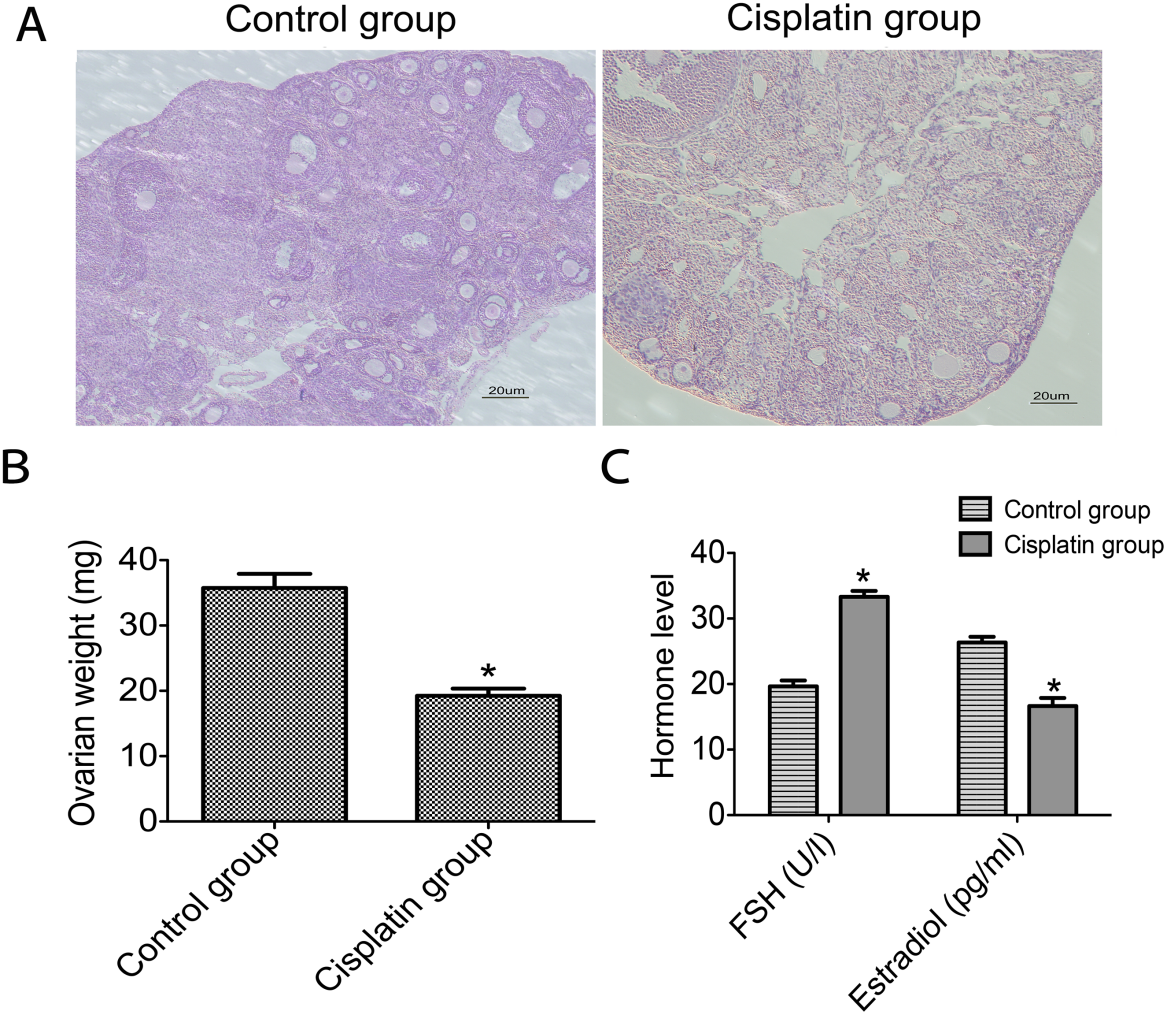
Morphologic change in ovarian tissues and hormone levels of the two groups. **A**: Morphology of ovarian tissues was determined by use of HE staining in both the control group and the cisplatin group (blue nuclei, red cytoplasm); **B**: Comparison of the ovarian weights; **C**: The comparison of hormone levels (FSH, Estradiol). *P<0.05 vs control group.

For detecting the correlation between Mfn2 and POF, we established a POF model in mice. The results show that cisplatin decreased the mice’s body weight, disrupted the estrous cycle, and significantly reduced ovarian weight (Fig 1B). We also noticed that within the ovarian tissues of the cisplatin group, there appeared stromal hyperplasia, antral follicle, and preantral follicles (Fig 1A). This of course differed from the control groups. Low levels of estradiol and elevated gonadotropin levels were observed in cisplatin groups (Fig 1C).

### Mfn2 levels are decreased in ovarian tissue

**Fig 2:**
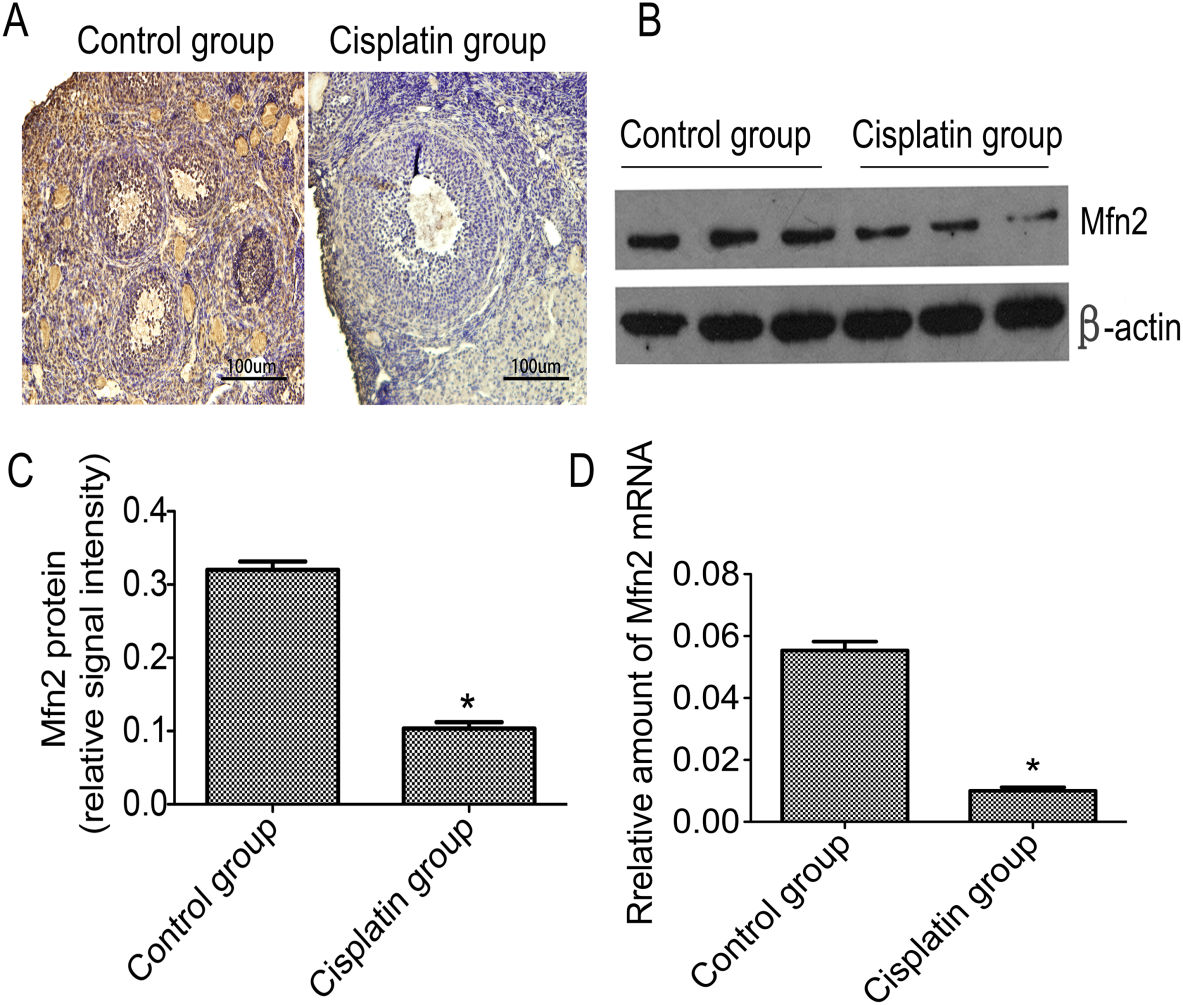
Mfn2 distribution and Mfn2 levels in ovarian tissues of the two groups. **A**: Mfn2 distribution was exhibited by immunohistochemistry and exclusively located in the cell's cytoplasm; **B**: The most representative image of western blotting; **C**: The relative amounts of Mfn2 protein levels in the control and cisplatin group; **D**: The comparison of Mfn2 mRNA levels in the ovarian tissue. *P<0.05 vs control group.

For detecting the distribution of Mfn2 in the ovarian tissues, we observed the expression of Mfn2 by immunohistochemistry. Results showed that Mfn2 was exclusively present in the cell's cytoplasm in both groups. However, a weaker immunostaining signal was observed in ovarian tissues of the cisplatin group relative to control (Fig 2A). We determined Mfn2 levels in the ovarian tissue by means of western blotting and q-PCR, in order to assess whether Mfn2 levels were different between the two groups. Results revealed that the relative amounts of Mfn2 in the cisplatin group were remarkably lower than the control group (Fig 2C). Protein loading from three different samples of each group are shown in Fig 2B. Q-PCR results confirmed the same findings (Fig 2D).

### Low expression of *Mfn2* is associated with apoptosis in POF

**Fig 3:**
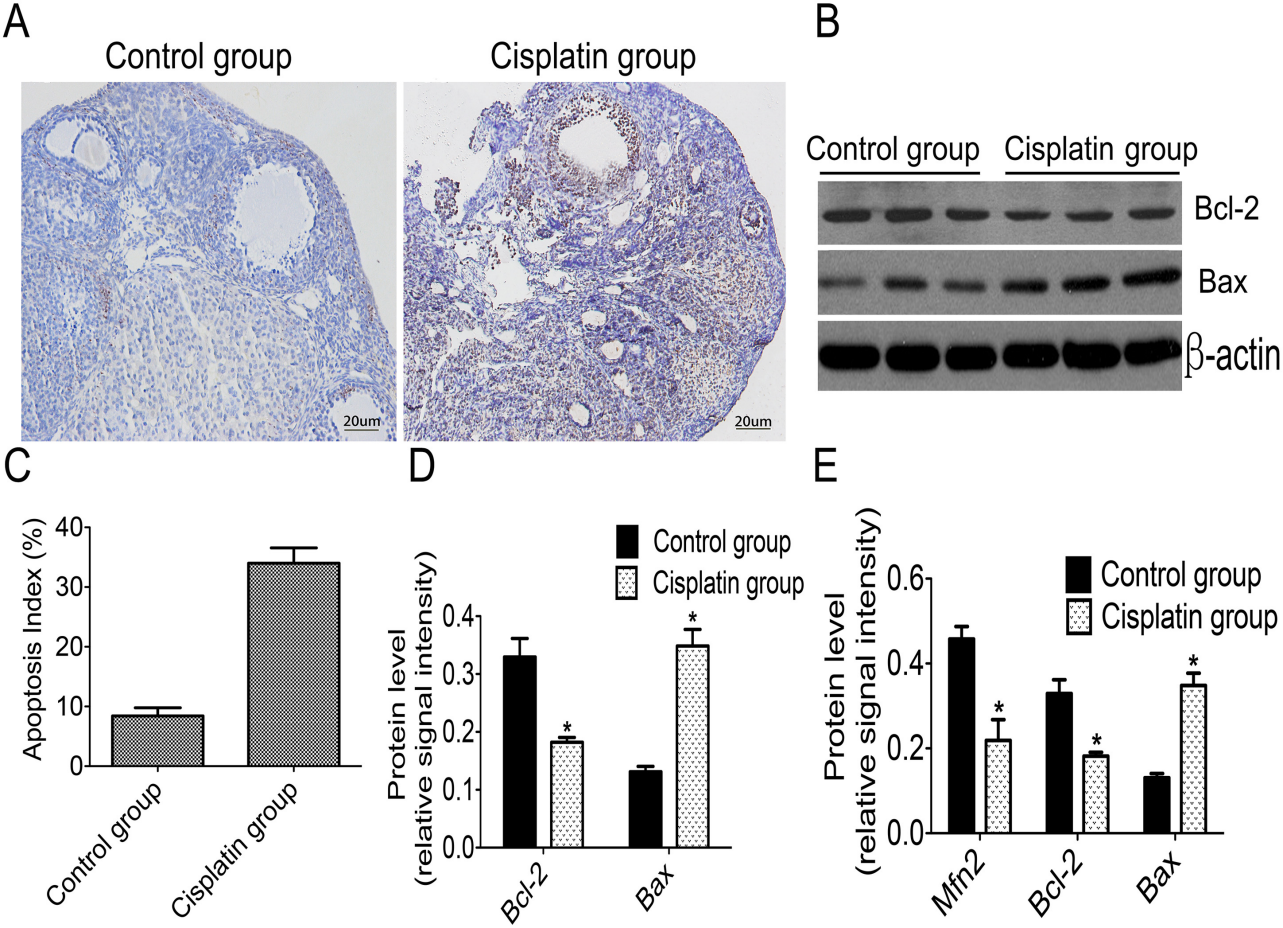
Low *Mfn2* expression is associated with cell apoptosis in mice's ovarian tissues of POF. A: Apoptosis was determined by TUNEL in the ovarian tissues (light staining in the cytotrophoblasts of the control group; deeper staining in the cisplatin group); **B**: The most representative images of western blot for Bcl-2 and Bax; **C**: Comparison of the Apoptosis Index in the ovarian cells; **D**: Comparison between Bcl-2 and Bax protein levels; **E**: Comparison of Mfn2, Bcl-2, and Bax protein levels. *P<0.05 vs control group.

We used TUNEL to detect cell apoptosis in finding the correlation between Mfn2 and cell apoptosis. Western blotting was used to analyze the expressions of anti-apoptotic protein Bcl-2 and apoptosis-promoting protein Bax. TUNEL-positive staining was observed in the ovarian tissue of both groups, and a light brown color was shown in samples from the control group (Fig 3A). However, a deeper brown color appeared in specimens from the cisplatin group (Fig 3A). The Apoptosis Index (AI) was 8.42%±2.35% in the control group and 34.01% ± 4.42% in the cisplatin group (Fig 3C) This suggests that the number of apoptotic cells in ovarian tissue from the cisplatin group was higher than that of the control group. In addition, Western blotting revealed that expression levels of Bax were increased in the cisplatin group (0.348±0.046) as compared with the control group (0.131±0.019) (Fig 3E). However, Bcl-2 was reduced in the cisplatin group (0.182±0.017) as compared to the control group (0.329±0.86) (Fig 3D). Protein loadings of three different samples from each group are shown in Fig 3B. One-way analysis of variance testing was used to address the correlation between Mfn2, Bcl-2 and Bax. Relative amounts of Mfn2 in the cisplatin group was positively correlated with Bcl-2, but negatively correlated with Bax levels (Fig 3E).

### Mitochondrial dysfuction is correlated with a reduction of Mfn2 levels in the ovarian tissue

**Fig 4:**
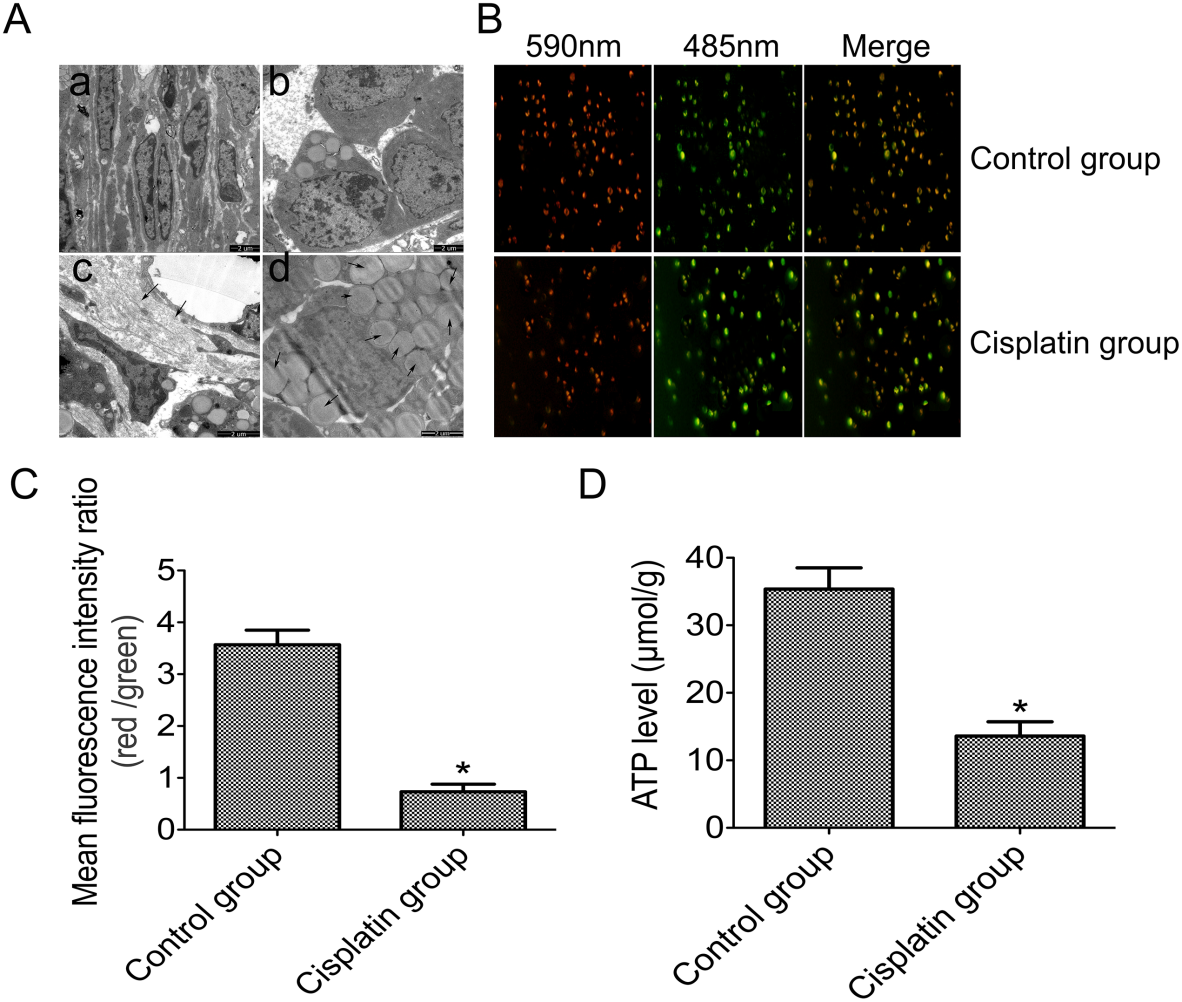
Mitochondrial dysfunction is correlated with reduced Mfn2 levels in ovarian tissue from the two groups. **A**: Ovarian tissue ultramicrostructure as detected by TEM (Aa: the most representative image of mitochondrial morphology and cristae in the control group; Ab: the most representative image of mitochondrial membrane and cristae in the cisplatin group; Ac: the fibrosis of ovarian tissues in the cisplatin group; Ad: the fat accumulation in ovarian tissues of the cisplatin group); **B**: The mitochondrial membrane potential (ΔΨm) as detected by using JC-1; **C**: Statistical analyses of the red and green fluorescence intensity ratios; **D**: ATP levels as determined by phosphomolybdic acid colorimetric method in the two groups. *P<0.05 vs control group.

In the cisplatin group, transmission electron microscopy (TEM) was used to detect the ovarian tissue ultramicrostructure in order to demonstrate the mitochondrial dysfunction in its ovarian tissue. There was a significant difference in mitochondrial morphologies between the two groups. In the control group, TEM revealed that mitochondrial morphology was intact and mitochondrial cristae still had a relatively regular arrangement (Fig 4Aa). However, in the cisplatin group, the mitochondrial membrane appeared impaired, and the mitochondrial cristae were arranged in a chaotic manner (Fig 4Ab). Ovarian fibrosis was also observed (Fig 4Ac), as was a significant accumulation of fat in the cells (Fig 4Ad).

We measured the ATP content of all the ovarian tissue samples by the phosphomolybdic-acid colorimetric method. Through JC-1, we linked mitochondrial membrane potential (ΔΨm) to mitochondrial function. We found a significant difference in the ΔΨm between the two groups: ΔΨm was significantly decreased (green) in the cisplatin group, as compared with the control group (red) (Fig 4B and 4C). At the same time, we found that the ATP content was decreased in the cisplatin group (13.5 ± 3.8 μmol/g), particularly when compared to the control group (35.2 ±5.7μmol/g) (Fig 4D). These results show that Mfn2 levels and mitochondrial function concurrently decrease.

## Discussion

Premature ovarian failure (POF) (also called primary ovarian insufficiency (POI) [23]) has an obvious influence on the fertility of women of child-bearing age. The difference between these women and menopausal women is that the ovaries in the former still contain a certain number of ovarian follicles. The general public has interest in how to keep these residual ovaries functioning, as well as improving their fertility. Therefore, it’s necessary to discover the causes and mechanisms of POF.

Studies have shown that injections of cisplatin (1.5mg/kg) in rats have an effect on ovarian function. This, in turn, can lead to a decline of the E_2_ level, an increase in levels of gonadotropin, disruption of the estrous cycle, and reduction of both the development and ovulation of oocytes [24]. Moreover, other reports have shown that cisplatin plays a vital role in the apoptosis of granulosa cells in follicles, which in turn can lead to follicular atresia [25,26]. Finally, our research has shown that intraperitoneal injections of cisplatin (1.5mg/kg) can successfully reconstruct the POF model. These studies have laid a foundation for further research in the correlation between Mfn2 and POF.

In recent years, more researchers are studying the etiologies and mechanisms of POF [2,3,6], which included the genetic contribution of the disease. Mfn2, a conserved dynamin-like GTPases that is located on the mitochondrial outer membrane and that is involved in the process of mitochondrial fusion, is conducive to the maximization of ATP production [27]. Moreover, Mfn2 also regulates functions of the endoplasmic reticulum's stress. It lastly regulates the mitochondrial function by inhibiting PERK pathways [28]. Studies showed that the lower expression of *Mfn2* could increase stress of the endoplasmic reticulum that results in the apoptosis of granulosa cells as well as impacting the synthesis of steroids [29,30]. With regards to the large amounts of energy dissipated in follicular development, we hypothesized that Mfn2 is involved in POF.

In our study, we observed the morphologic and structural changes in ovarian tissues and the distribution of Mfn2 within them. We found that body weight declined, that the estrous cycle was disordered, and that the ovarian weight was significantly reduced in the cisplatin group. When compared with the control group, we also found that in the ovaries stromal hyperplasia appeared, antral follicles appeared, and preantral follicles decreased. Most importantly, low levels of estradiol and elevated levels of gonadotropin were observed in the cisplatin groups. We found that the level of *Mfn2* in the cisplatin groups were remarkably lower than those in the control group and that, moreover, it was localized exclusively in the cell's cytoplasm. Overall, our study suggests that the inadequate expression of *Mfn2* in ovarian tissues of mice probably has a close relation with the change of ovarian morphology and function in POF.

The successful development of follicles is dependent on various factors that include the coordinated expression between receptors and hormones, cytokine stimulation, optimal PH, and sufficient energy supply. The latter is particularly important, as mitochondria produce adenosine triphosphate (ATP), and ATP is the main energy provider for life. Therefore, mitochondria play a vital role in follicular development and survival.

The connection between mitochondria and the endoplasmic reticulum affects the stability of intracellular calcium and cell apoptosis [31]. Fusion and fission are constantly performed by mitochondria, and these processes are particularly important in maintaining normal mitochondrial morphology. In addition, a disordered mitochondrial metabolism is mainly characterized by the reduction of ATP, the decline of mitochondrial membrane potential, and the increase in apoptosis. The ROS and stress response lead to these three processes [32]. A previous study suggested that a lower ATP levels effect follicular development and oocyte’ maturation [33]. However, on the other hand, excessive production can also result in irreversible damage to the mitochondria, as excessive free radicals leads to apoptosis of the oocytes as well [34]. As one of the most important proteins involved in mitochondrial dynamics, Mfn2 plays an integral role in regulating mitochondrial morphology and maintaining the stability of mitochondrial DNA. Researches have revealed that Mfn2 is involved in the regulation of the mitochondrial metabolic pathways in the development processes of both oocytes and preimplantation embryos [20,35]. In our study, we found that the ATP content was noticeably decreased in the cisplatin group (as compared with control group) and that this, was accompanied by a decreased level of Mfn2 and a change of mitochondrial morphology. Thus, we presume that Mfn2 potentially participates in disordering the development of follicules, and thus in turn, by regulating mitochondrial function and energy metabolism, leads to POF.

Apoptosis is an ATP-dependant process [36,37]. It has been shown that the apoptosis of ovarian granular cells and oocytes is the main factor in the occurrence of POF [2,3]. Several apoptotic pathways have been reported, including the mitochondrial, the death receptor, and the endoplasmic reticulum pathway [38]. In mammalian cells, the mitochondrial pathway is the most common apoptotic mechanism and constitutes the core part of the pathway [39]. The anti-apoptotic protein Bcl-2, located in the mitochondria, maintains the integrity of the mitochondria and hinders the release of cytochrome C, however, the apoptosis-promoting protein Bax promotes the entire process [40].

When compared with the control group, we found that the Apoptosis Index (AI) of ovarian tissues in the cisplatin group was markedly higher. In addition, the expression levels of *Bcl-2* were reduced in the cisplatin group, as were levels of *Mfn2*, while levels of *Bax* were increased. Previous reports of *Mfn2* address that *Mfn2* may in fact promote apoptosis by affecting the expression of *Bcl-2*, the *Bax* protein family, the release of cytochrome C, and the activation of caspase. So we speculate that Mfn2 potentially take parts in the pathogenesis of POF via apoptotic balance, itself controlled by Bcl-2/Bax.

A normal mitochondrial membrane potential (ΔΨm) is essential in maintaining the respiratory chain and Ca^2+^ metabolic regulation. However, should these be changed, many effects can occur, including the leakage of protons, the opening of the transformational aperture on the mitochondrial membrane, and the release of cytochrome C. These three effects, eventually, all cascade in a reaction that results in apoptosis. In our study, we found that ΔΨm was significantly decreased in the cisplatin group. Furthermore, by TEM, we found that the ultramicrostructure accumulated a significant amount of fat. This indirectly hints the decay of mitochondrial function. As such, our results further suggest that inadequate levels of Mfn2 in ovarian cells have a close relationship with the occurrence of POF.

In summary, our results reveal that expressions of *Mfn2* are decreased in the ovarian tissues of POF, and that fact may be a mechanism involved in both mitochondrial damage and an increase in apoptosis. Therefore, we devote ourselves to further research that addresses the mechanism of upstream signal conditioning of Mfn2, and explores the relationship between Mfn2 and the mitochondrial apoptotic pathway.
